# How much consciousness is there in complexity?

**DOI:** 10.3389/fpsyg.2022.983315

**Published:** 2022-09-20

**Authors:** Marcin Koculak, Michał Wierzchoń

**Affiliations:** ^1^Consciousness Lab, Institute of Psychology, Jagiellonian University, Kraków, Poland; ^2^Centre for Brain Research, Jagiellonian University, Kraków, Poland

**Keywords:** consciousness, complexity, neural correlates of consciousness, state, content

## Abstract

The notion of complexity currently receives significant attention in neuroscience, mainly through the popularity of the Integrated Information Theory (IIT). It has proven successful in research centred on discriminating states of consciousness, while little theoretical and experimental effort was directed toward studying the content. In this paper, we argue that exploring the relationship between complexity and conscious content is necessary to understand the importance of information-theoretic measures for consciousness research properly. We outline how content could be experimentally operationalised and how rudimental testable hypotheses can be formulated without requiring IIT formalisms. This approach would not only allow for a better understanding of aspects of consciousness captured by complexity but could also facilitate comparison efforts for theories of consciousness.

## Introduction

The notion of complexity currently receives a significant amount of attention in neuroscience. It is frequently used as a shorthand for applying the information-theoretic approach to study the relation between the mind and the brain. Although problematic (Ladyman et al., 2013), this could be seen as an extrapolation of the complexity of the brain as a biological structure to the mind. It could also be an inevitable consequence of the dominance of the computer metaphor in cognitive neuroscience (Gigerenzer and Goldstein, 1996), where the mind is conceptualised as an information processing system. Similarly, in consciousness science approaches based on complexity were first popularised by the work of Tononi and Edelman (1998) and later evolved into Integrated Information Theory (IIT; Oizumi et al., 2014), which is considered presently one of the most influential theories available. A large part of this prominence can be attributed to the successful application of complexity measures to the discrimination of states of consciousness (Sarasso et al., 2021), supporting the general theoretical claims of the theory.

Complexity became so ubiquitous in the literature about IIT that it is frequently treated synonymously with applying the theoretical principles of this approach. This limits the discussion on the usefulness of information theory in understanding the neural basis of consciousness to the ontological frame of IIT. Consequently, critical assessments of IIT tend to dismiss the usefulness and importance of complexity measures based on theoretical problems that IIT bears in their view (for a recent example, see: Merker et al., 2022; with responses). The impracticality of this situation has been recently recognised by Mediano et al. (2022); however, they advocate only on behalf of a “weak version” of IIT with relaxed ontological claims, focused on a more thorough assessment of the behaviour of measures of information dynamics to different aspects of consciousness. In this article, we call for a more radical decoupling of the notion of complexity from IIT, allowing for a broader assessment of its usefulness irrespective of the theoretical approach. Furthermore, we argue that every theory employing complexity measures must address its relation to conscious content to be treated as a proper theory of consciousness.

## Paradox of phenomenology in integrated information theory

The proponents of IIT describe it as derived solely from the phenomenology of conscious experience (Tononi et al., 2016). They argue that philosophical analysis of the structure of phenomenal experience, translated into physical terms, creates an identity relation where all of the subjectivity is captured through the properties of a conceptual structure. Moreover, this precise translation based on mathematical notations should allow, in principle, scientific inquiry of said structure. This theoretical assumption equates investigation of its properties to exploring subjective experience, legitimising the use of numerical methods as indicators of the presence of consciousness as such. Since this structure is defined in terms of cause-effect power as a multiway interaction of simple elements (Oizumi et al., 2014), it can be assessed, or at least approximated, by measures of complexity (Arsiwalla and Verschure, 2018). IIT interprets these measurements as describing the quantitative aspect or consciousness (Tononi and Koch, 2015), which in empirical studies is equated to the state of consciousness (Sarasso et al., 2021), spanning from full wakefulness to deep sleep, anaesthesia or coma.

Proponents of IIT point to this line of reasoning as an argument in favour of the validity of the whole theory. They start from the core of consciousness, namely phenomenal experience. Through logical and mathematical analysis, they arrive at a conceptual model, parametrisation of which allows measuring consciousness in real-world data. It seems, however, that this completeness is only illusory since the notion of consciousness they started with differs from the one identified at the end. IIT begins with formulating five axioms about the phenomenal experience (Oizumi et al., 2014) that refer only to its formal properties. Importantly, these axioms seem to operate in an all-or-nothing manner, being strictly necessary for the subjective experience to arise. On the other hand, what is typically assessed in studies employing complexity measures is only the state of consciousness (Sarasso et al., 2021), often interpreted rather as a general level of wakefulness. It is typically thought of as a continuum with different levels (Bayne et al., 2016), but it is not derivable from axioms proposed by IIT as it does not refer directly to any phenomenal properties. Interestingly, neither the formal analyses nor the empirical studies include the notion of conscious content, which is the central focus of phenomenology and a necessary element for ascribing consciousness to a person.

There is no denying that measures derived from information theory have proven to be a robust indicator of the level of consciousness in clinical and non-clinical conditions (Sarasso et al., 2021), also in comparison with other approaches (Nilsen et al., 2020). Though these conditions are characterised by disparate differences between consciousness and unconsciousness, with accompanying profound physiological changes, proponents of IIT tend to treat this as evidence confirming the theory’s assumptions. However, with the lack of studies tying derived measures to phenomenal qualities of conscious content, opponents of this approach can always point to the relation between the state and complexity as an argument against identifying it with actual consciousness. They could argue that this observed relation indicates that complexity measures capture only the necessary but not sufficient properties for conscious awareness. Merker et al. (2022) name it “efficient information processing” and suggest it might be a general organisational property that by design or evolution can be found in many complex systems, most of which can hardly be described as conscious or even alive in any meaningful way.

Interestingly, referring to axioms as a direct connection of the theory to phenomenology could also be treated as necessary but insufficient. Since the properties of the abstract causal structure are based on axioms derived from structural aspects of experience and do not describe the phenomenal content itself, they too can be interpreted as necessary but not sufficient for consciousness. What is more problematic, the way axioms are formulated prevents any form of experimental manipulation that could prove their sufficiency. Paradoxically, distilling the phenomenality of conscious experience in IIT to its fundamental properties might have led to axioms that are not specific enough to constitute the conscious subjective experience. Therefore, it seems crucial for IIT or any approach based on complexity to explore the relationship between those measures and conscious content experimentally, as this is the only way to prove that it captures the neuronal basis of consciousness and not only something like efficient information processing.

## Complexity and conscious experience

One of the biggest appeals of IIT is its promise to capture in a single mechanism both dimensions typically used to describe consciousness, namely the state and content (Tononi and Koch, 2015). The state is understood quantitatively as the degree of integration, and a special measure Φ is designed to represent it. On the other hand, the content is described as the shape of this conceptual structure, but currently, IIT does not provide any way of quantifying it. Some researchers point to the rate of change of conscious experience in time as its measurable aspect and connect it to the concept of differentiation (Sarasso et al., 2021). This seems surprising as most theoretical foundations of IIT are built upon “phenomenological atoms” that constitute the conceptual structure in its core and are organised according to proposed axioms. This can be seen in graphical representations accompanying theoretical analyses, where each structural element is a distinguishable phenomenal quality (Tononi et al., 2016) matched to a particular set of neurons. In newer works, in which proponents of IIT attempt to formally describe the experience of space (Haun and Tononi, 2019; Ellia et al., 2021), they seem to go even further, dividing space into small parts (akin to pixels in an LCD screen) that can be related structurally and functionally to the organisation of neurons in the cortex. Conscious experience in IIT has an inherent quantitative granularity built in that has not been yet translated into testable predictions, although these space-related papers seem like a groundwork for future experimental studies.

This situation is understandable since the focus of IIT is on the mechanism of integration, where the whole conceptual structure representing subjective experience exceeds the contribution of its parts and constitutes consciousness as such. This emergent behaviour being a central part of the theory is probably one of the reasons some researchers think that a weaker version, not tied to particular ontological claims, would allow for more broad research (Mediano et al., 2022), benefiting in the end, the IIT itself. We agree that loosening theoretical ties between IIT and measures based on information theory would accelerate the assessment of their connection to consciousness. However, we think that a more radical decoupling is necessary to make the best use of the research resources available now. In the rest of the paper, we want to propose how testable predictions about the conscious experience can be formulated that take advantage of the robustness of complexity measures and are also based on fundamental phenomenological properties but avoid strong ontological claims of any particular theory through relying only on assumptions present in the general paradigm of cognitive neuroscience that overarches most of the contemporary theories of consciousness. This common denominator of treating the brain and mind as information processing systems lines itself well with information-theoretic measures, allowing for a common ground on which different theories could be compared and evaluated.

## Complexity and conscious content

In our view, the central phenomenological insight connected to the notion of complexity is the richness of conscious experience (Block, 1995). In the most general sense, it refers to a plethora of content populating subjective experience that is clearly distinguishable and has various qualitative properties. The extent of this richness is still being debated (Kouider et al., 2010; Block, 2011), but it is hard to deny that during normal wakefulness, a person is simultaneously consciously aware of multiple things, e.g., objects present in their field of view. The second insight concerns the unity of conscious experience, but only to the extent that all of conscious content is combined and arranged in one coherent entity. Crucially, this entity feels complete and fully occupying the “space in our mind,” yet trivial examples prove that we can meaningfully describe it in quantitative terms, e.g., closing one’s eyes or turning off the radio lowers the number of things one is conscious of.

Moreover, content not only coexists in conscious experience, but also all elements are in relation to each other, creating a complex arrangement that is more than just the sum of its parts. This, of course, echoes the views of the Gestalt tradition (Wertheimer, 1938) but also is in line with the IIT as it comes to richness (Haun et al., 2017) as well as unity, which is one of the axioms (Oizumi et al., 2014). Assuming every experienced content and its qualities have some distinct neural basis, we can provisionally postulate that interactions between contents of consciousness should be reflected by some neural processes. Therefore, richness of conscious experience would have to correlate with some aspects of the complexity of neuronal interactions (e.g., local or global dynamics, non-linear causal influence, or their interaction in a form of hierarchy of complexity).

Following this line of thought, we can formulate testable hypotheses based on the assumption that some aspect of phenomenal experience, namely conscious content, can be quantified and experimentally manipulated to search for brain activity correlated with those subjective changes. Most intuitively, this quantification of content can be understood in a straightforward additive sense, e.g., there is more conscious content when a participant is presented with two objects on the screen instead of only one. The rationale would point to the engagement of more sensory neurons for longer periods, creating a more complex interaction between them and other cortical regions. Similarly, this would also include variation in the intensity of physical stimulation, e.g., brightness or loudness, that results in changes in the experience. This psychophysical approach would allow for fine-grained control over participants’ subjective experience, enabling the researchers to search for a measure that would generalise over different qualities and modalities. Crucially, comparisons would be made when subjects are fully awake, ensuring that variability in the state of consciousness is minimised.

Alternatively, one can point to multisensory integration as another way for one’s subjective experience to be richer. For example, presenting a movie snippet with synced or misaligned video and audio tracks can be interpreted as addition of perceptual but not physical quality that makes the synced material richer for the participant. This could also be extrapolated on concepts like temporal integration, where the proper order of stimulation, for example a sequence of scenes in a play, allows for a more informationally rich experience. Treating this as an experience contextualised in time, we can also speculate that a similar effect could be observed in the spatial domain. The obvious examples would include laws of perception proposed by the Gestalt school (Wertheimer, 1938) or visual illusions, where specific placement of elements generates more perceptual experience than is present in physical stimulation alone, e.g., Kanizsa triangles (Kanizsa, 1987). Similarly to the psychophysical manipulation mentioned earlier, the state of consciousness is kept constant, but here also the physical stimulation is the same. Despite that, one of the conditions seems to have more qualities than the other.

Experimental support for this line of reasoning already exists. Some of the studies following these principles were conducted by the proponents of IIT themselves. A paper by Boly et al. (2015) assessed the complexity of brain activity recorded with fMRI in response to a short movie, the same movie but with parts in random order, or a static TV noise. They reported an increased level of complexity, as measured by Lempel-Ziv compressibility, from the noise condition through scrambled to the movie in the proper sequence. Importantly, in the general sense, participants maintained the same level of consciousness throughout the whole experiment, so it is reasonable to assign the effects to changes in the content. Interestingly, it seems that not only do we observe an increase in complexity through adding the number of objects (no discernible objects in noise versus movie frame full of content), but also through the introduction of meaning stemming from watching the movie in proper sequence.

There is also a handful of similar effects reported for speech perception (Borges et al., 2018), music production and reception (Dolan et al., 2018), tracking meaningfulness of images (Mensen et al., 2017) and video clips (Mensen et al., 2018), or bistable perception (Canales-Johnson et al., 2020). However, some studies did not find significant differences in similar paradigms (Bola et al., 2018). Sparsity of experimental evidence, mostly small sample sizes, and vastly different paradigms used, indicate a striking disproportion in the amount of attention devoted to studying content compared to states of consciousness. This might result from differences in the magnitude of effects, making it significantly more challenging to show the relation between complexity and conscious content systematically. Despite that, if information-based approaches want to make a compelling case about the mechanism of consciousness, they need to reliably demonstrate how variation in content is accompanied by changes observed through measures of complexity.

We are aware that readers of this article might find the parallel between richness of phenomenal experience and complexity of neuronal interactions as superficial and naïve, a case of mistaken identity, similarly to the critique IIT is facing (Merker et al., 2022). However, we are convinced that consciousness science can only benefit from systematic experimental research that expands beyond the narrow definition of conscious content as isolated objects presented briefly on a monitor’s screen. Although simplistic, the proposed approach introduces a principled way in which subjective experience can be experimentally manipulated with more naturalistic, complex, meaningful stimulation. Combining it with complexity measures, currently the most robust tools for detecting conscious activity (Nilsen et al., 2020), gives us a set of testable predictions that even if proven wrong, will expand our understanding of the relation between consciousness and the brain activity.

It is essential to acknowledge that although our approach is deliberately broad enough not to be bound by a conceptual framework of a particular theory, it is still rooted in a research paradigm that seeks for neuronal activity to explain consciousness. While being the most widespread approach among consciousness research community, there are other options available (e.g., Dennett, 1993; Frankish, 2016; Schurger and Graziano, 2022), where phenomenality is denied importance. Some researchers (Rahimian, 2022) argue for their importance as only a radical shift in our conceptualisation of consciousness of a similar kind could move science forward. Our proposition is not aimed at improving the existing frameworks to exceed the limitations of their paradigm. We rather hope for pushing the available methods and theories to their logical limit and hopefully introducing more “points of contact” for the theories to be compared and evaluated. Therefore it must function in the general frame of the paradigm and can be subjected to the same criticism that theories it shares the assumptions with.

## Moving forward

There is no denying that complexity captures an essential aspect of brain activity closely related to consciousness. It reliably dissociates levels of wakefulness and shows some promise to quantify the “amount” of phenomenal experience people have. There are, however, many unknowns related to proper ways of calculating the measures of complexity, decisions about the spatial and temporal scale they should be applied to, picking the optimum level of neural hierarchy to assess, or properly defining the conditions that should be contrasted (Sarasso et al., 2021). In our view, progress in these areas is hampered by the connection of the concept of complexity to only one specific theory and treating the results acquired with it as a confirmation of the theoretical assumptions that IIT is founded on. Its critics frequently point to research arguing that similar results could be obtained by many different architectures and systems (Doerig et al., 2019), but similarly treat it only as an argument against IIT and not as the authors intended—a challenge to a whole research program shared by many theories. While many researchers are not convinced that a new paradigm is necessary, there are still new directions we can take to make current efforts more robust and valuable in understanding consciousness.

The most obvious first step would be to systematically explore the relationship between measures of complexity and variability of states and contents of consciousness in broad spectrum of experimental data. Importantly, explicit manipulation of the conscious content is necessary to make any claims about capturing the phenomenal aspect of the experience. This could be realised in several ways, e.g., utilising resting-state paradigms where participants are passively exposed to stimulation on different levels of complexity (Koculak and Wierzchoń, 2022). This would allow for selectively manipulating and comparing the amount of information introduced in one modality and introducing conditions with increasing multimodal complexity. Additionally, using more naturalistic stimuli that imitate real-world experience should make the differences between these conditions more pronounced than artificially generated distortions.

Another option would be tapping into the existing plethora of experimental data, where different aspects of the conscious experience were manipulated and analysed in the context of various theories of consciousness (Yaron et al., 2022). Assuming complexity tracks crucial aspects related to conscious processing, it should be able to discern conscious perception from the unconscious, e.g., in an experiment manipulating awareness of backward masked visual stimuli. Mensen et al. (2017) do it for a novel paradigm, but there is no principled reason why similar analyses could not be done on other already published data. This would have the added benefit of the possibility of comparing how complexity analysis relates to methods like ERPs in capturing changes in consciousness. Collecting a significant amount of such comparisons should highlight aspects where methods agree and disagree, potentially guiding new research paradigms that would allow for a more rigorous comparison of theories (Del Pin et al., 2021; Melloni et al., 2021).

## Data availability statement

The original contributions presented in this study are included in the article/supplementary material, further inquiries can be directed to the corresponding author.

## Author contributions

MK drafted the initial version. Both authors revised the manuscript.
